# Bronchial microdialysis monitoring of inflammatory response in open abdominal aortic aneurysm repair; an observational study

**DOI:** 10.14814/phy2.13348

**Published:** 2017-07-25

**Authors:** Stig S. Tyvold, Torbjørn Dahl, Stein Dragsund, Sigurd Gunnes, Oddveig Lyng, Jan K. Damås, Petter Aadahl, Erik Solligård

**Affiliations:** ^1^ Department of Circulation and Medical Imaging Norwegian University of Science and Technology Trondheim Norway; ^2^ Clinic of Surgery St. Olavs Hospital Trondheim Norway; ^3^ Clinic of Anesthesia and Intensive Care St. Olavs Hospital Trondheim Norway; ^4^ Clinic of Cardiothoracic Surgery St. Olavs Hospital Trondheim Norway; ^5^ Faculty of Medicine Norwegian University of Science and Technology Trondheim Norway; ^6^ Centre of Molecular Inflammation Research Department of Cancer Research and Molecular Medicine Norwegian University of Science and Technology Trondheim Norway; ^7^ Mid‐Norway Sepsis Research Group NTNU and St. Olavs Hospital Trondheim Norway

**Keywords:** Aortic surgery, cytokines, lung, reperfusion injury

## Abstract

Aortic surgery results in ischemia–reperfusion injury that induces an inflammatory response and frequent complications. The magnitude of the inflammatory response in blood and bronchi may be associated with the risk of immediate complications. The purpose of the study was to evaluate bronchial microdialysis as a continuous monitoring of cytokines in bronchial epithelial lining fluid (ELF) and to determine whether bronchial ELF cytokine levels reflect the ischemia–reperfusion injury and risk for complications during open abdominal aortic aneurysm (AAA) repair. We measured cytokines in venous blood using microdialysis and in serum for comparison. Sixteen patients scheduled for elective open AAA repair were included in a prospective observational study. Microdialysis catheters were introduced into a bronchi and a cubital vein. Eighteen cytokines were measured using a Bio‐Plex Magnetic Human Cytokine Panel. Samples were collected before and during cross‐clamping of the aorta as well as from 0 to 60 min and from 60 to 120 min of reperfusion. The ELF levels of several cytokines changed significantly during reperfusion. In particular, IL‐6 increased more than 10‐fold and IL‐13 more than 5‐fold during ischemia and reperfusion. Also, the venous levels of several inflammatory and anti‐inflammatory cytokines increased and exhibited their highest concentration during reperfusion. Both bronchial and venous cytokine levels correlated with duration of the procedure, intensive care days, and preoperative kidney disease. Three patients suffered organ failure as a direct consequence of the procedure, and in these patients the bronchial ELF concentrations of 17 of 18 cytokines differed significantly from patients without such complications. Bronchial microdialysis is suited for continuous monitoring of inflammation during open AAA repair. The bronchial ELF cytokine levels may be useful in predicting immediate complications such as organ failure in patients undergoing vascular surgery.

## Introduction

Patients undergoing major vascular surgery are prone to complications such as peri‐ and postoperative respiratory and renal failure. The estimated risk of any organ failure after elective open infrarenal aortic repair varies from 16% to 36% (Roumen et al. 1993; Muehling et al. 2011). It is a major challenge to identify patients at risk for a complicated course. Besides patient characteristics such as smoking, diabetes, obesity, and renal failure, perioperative measures such as duration of the procedure are predictors for complications during vascular surgery. Several studies have shown that an enhanced inflammatory response during surgery seems to reflect an increased risk for complications in these patients Thus, elevated cytokine levels have been associated with respiratory failure and increased mortality in patients undergoing aortic surgery (Roumen et al. 1993; Cornet et al. 2009).

Microdialysis is a minimally invasive technique used to explore tissue chemistry within target organs and has gained attention in studies on pharmacokinetics of antibiotic drugs in the lungs, as well as a valid “bedside” method to detect acute ischemia and in measurements of inflammatory markers that predict organ rejection in transplantation surgery (Herkner et al. 2002; Aoki et al. 2008; Nielsen et al. 2011; Haugaa et al. 2012).

We have previously shown that detection of substances in the epithelial lining fluid (ELF) by bronchial microdialysis (BMD) is superior to measurements in serum samples for detecting inflammation in a pig model of intestinal ischemia–reperfusion (IR) injury, which suggests that bronchial cytokine concentrations could be a warning sign and a criterion to expedite clinical decision making (Tyvold et al. 2007, 2010).

In the present study, we hypothesize that cytokine levels in bronchial ELF may differ from the levels in systemic circulation, and that BMD may be a method useful in monitoring pulmonary inflammatory responses and thereby identifying patients at risk for complications. To explore this hypothesis, we studied patients undergoing elective open abdominal aortic aneurysm (AAA) repair and used microdialysis catheters positioned in a bronchus and a cubital vein for obtaining samples for cumulative cytokine analyses both peri‐ and postoperatively.

## Materials and Methods

The protocol was approved by the Regional Committee for Health and Medical Research. Informed consent was obtained from each patient. The study is registered at clinicaltrials.gov (Tyvold 2011). Sixteen patients scheduled for elective open AAA repair at St. Olavs Hospital, Trondheim University Hospital were included in this prospective observational study. No patients were excluded. Morbidity and mortality were registered 30 days postoperatively. Patient characteristics are presented in Table 1.

**Table 1:** Patient characteristics during the 30 days observation period

	Non organ failure (*n* = 13)	Organ failure (*n* = 3)	***P***
	Median (min–max)	Median (min–max)	
Age (years)	70 (61–83)	71 (65–76)	1.000
Height (cm)	180 (167–197)	175 (175–178)	0.146
Weight (kg)	86 (78–120)	105 (76–121)	0.611
Body mass index	28 (24.8–34.7)	38.2 (24.8–38.2)	0.439
ASA	3 (2–3)	3 (3–3)	0.529
Aortic aneurysm diameter (mm)	60 (54–86)	63 (60–78)	0.364
s‐Creatinine	82 (58–166)	100 (99–166)	0.082
Glomerular filtration ratio	68 (53–90)	51 (51–71)	0.146

	Number of patients	Number of patients	***P***
Hypertension	7 of 13	1 of 3	0.500
Coronary arterial disease	7 of 13	2 of 3	0.600
Coronary intervention	6 of 13	2 of 3	0.500
Heart failure	4 of 13	0 of 3	0.393
Other vascular disease	5 of 13	0 of 3	0.295
COPD	0 of 13	0 of 3	–
Renal disease	1 of 13	1 of 3	0.350
Liver disease	0 of 13	1 of 3	0.188
Diabetes mellitus	2 of 13	0 of 3	0.650
Smoking	7 of 13	2 of 3	0.600
Statin use	10 of 13	3 of 3	0.511
Acetylsalicylic acid use	10 of 13	3 of 3	0.511

	Number of procedures	Number of procedures	*P*
Straight graft	8	2	
Y‐graft	5	1	0.696

	Median (min–max)	Median (min–max)	*P*
Clamp time (hh:mm)	01:12 (00:48–01:49)	02:02 (01:07–02:16)	0.146
Surgery (hh:mm)	03:20 (01:51–04:58)	04:21 (02:21–04:58)	0.521
Anesthesia (hh:mm)	05:05 (02:48–06:45)	05:35 (04:10–06:45)	0.296
Bleeding (mL)	1390 (995–2930)	4500 (380–5960)	0.439
Erythrocyte transfusion (mL)	480 (0–1000)	1000 (500–1000)	0.057
Auto transfusion (mL)	426 (178–1093)	1352 (150–2912)	0.439
Other IV fluids (mL)	6490 (3600–12000)	7900 (2600–8700)	0.704
Diuresis (mL hour^−1^)	1 (0.4–3.5)	0.6 (0.2–1.9)	0.521
Fluid balance (mL)	4902 (2743–9790)	5742 (2670–7396)	0.800
Hospital stay	7 (5–39)	11 (10–27)	0.189
Intensive care unit days	0 (0–13)	5 (1–13)	0.057
High dependency unit days	4 (1–22)	5 (5–6)	0.611
Surgical ward days	4 (1–17)	3 (3–8)	0.364
Organ failures within 30 days	1 (0–3)	2 (2–3)	0.146

Patients with organ failure triggered only by the trauma of the AAA surgery procedure, according to the organ failure criteria (Supporting Information), persisting more than 24 h, and required treatment and observation in the intensive care unit, were included in the primary postoperative *organ failure* group (Table 2a). Patients without complications, with transient organ failures (less than 24 h, not leading to intensive care therapy) and patients with complications, like bleeding and aspiration, occurring on the first postoperative day and later, leading to reoperation and/or intensive care, were not included in the organ failure group (Table 2b).

**Table 2:** Registered complications

Complication	Treatment/Course	Intensive care unit
(a) Description of complications included in the organ failure group (3 of 16 patients)		
Circulatory instable during surgery. Fluid and norepinephrine dependent	Norepinephrine >10 *μ*g/kg/h, supportive mechanical ventilation, and critical care treatment	11 days
Postoperative renal failure (s‐creatinine max 195 mmol/L)	Acute renal failure not requiring dialysis. Intermittent supportive mechanical ventilation	5 days
Peri‐ and postoperative circulatory failure and acute or chronic renal impairment	Norepinephrine >10 *μ*g/kg/h, fluid treatment, and supportive mechanical ventilation	1 day
(b) Description of short‐term and late complications in the non‐organ failure group (6 of 16 patients)		
Aspiration of gastric contents into the lungs on ward, postoperative day 2	Chemical pneumonitis requiring mechanical ventilation	13 days
Transient postoperative hypoxia	Intermittent CPAP <24 h postoperatively	0 days
Reopened after closure, before leaving the operation theater, because of negative Doppler signal in lower extremities	Norepinephrine infusion <10 *μ*g/kg/h	0 days
Resuture of distal anastomosis due to leakage before closure in operation theater	Frequent ventricular extrasystole. Norepinephrine infusion <10 *μ*g/kg/h	0 days
Late postoperative urosepsis	Late acute renal failure not requiring dialysis and CPAP treatment	0 days
Reoperated because of bleeding on postoperative day 1. Reoperated 14 days later because of intestinal perforation	After the second reoperation, supportive mechanical ventilation pneumonia, norepinephrine >10 *μ*g/kg/h and late acute renal failure not requiring dialysis	7 days

### Anesthesia and surgery

Standard open AAA repair was performed during general anesthesia and thoracic epidural analgesia, by a midline laparotomy, and cross‐clamping of the abdominal aorta below the renal arteries with the insertion of a tubular or bifurcated graft.

### Microdialysis and blood samples

All microdialysis catheters were tested, connected to microdialysis pumps (CMA 107, CMA Microdialysis AB, Stockholm, Sweden), and continuously perfused 1 h before and during insertion using a sterile infusion (Plasmodex^®^, Meda AB, Solna, Sweden) with a flow rate of 1 *μ*L/min (Tyvold et al. 2010). Microdialysis vials (Microdialysis AB, Sweden) were labeled and weighed before and after sample collection. The density of Plasmodex^®^ (Meda AS, Asker, Norway) differed <1% from water and was assumed to be 1 mg/*μ*L. The mean fluid recovery was 94% in venous microdialysis (VMD) and 88% in BMD.

The catheters were perfused in situ 5–10 min before sampling. The samples were immediately stored at −80°C. The microdialysis catheters were removed within minutes after completion of the experimental protocol 120 min after the start of reperfusion.

### Bronchial microdialysis

**Figure 1:**
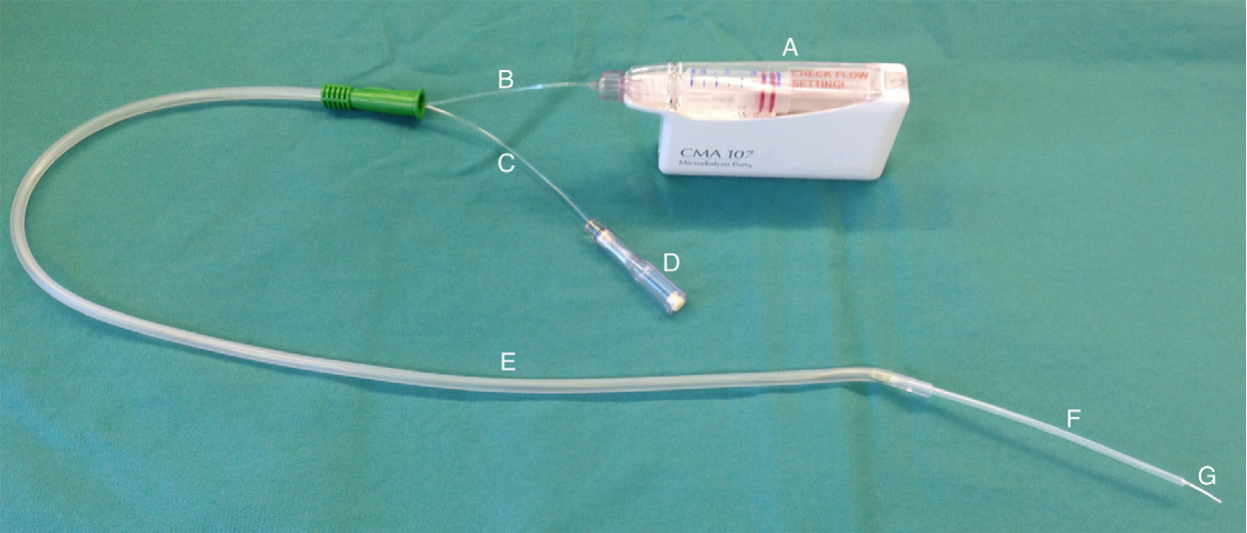
The bronchial microdialysis catheter prepared for insertion. (A) Microdialysis pump, (B) inlet, (C) outlet, (D) collection vial, (E) 14G suction catheter, (F) protection sheath, cut to fit, (G) semipermeable membrane, 10 mm.

A microdialysis catheter (CMA 71, custom made, cutoff 100 kDA, membrane length 10 mm, CMA Microdialysis AB, Stockholm, Sweden) was threaded into a 14‐G suction catheter before insertion (Fig. 1). The microdialysis catheter was introduced into the trachea under direct laryngoscopy before endotracheal intubation, the catheter was advanced until it wedged in the bronchial tree, after endotracheal intubation, fiber‐optic bronchoscopy was performed to verify the wedged position. Patients were observed for signs of complications related to the microdialysis catheters, including bronchial reactivity requiring antiobstructive medication, bronchial bleeding, pneumothorax, and pneumonia. The catheters were inspected for damage after removal.

### Intravenous microdialysis

The microdialysis catheter (CMA 71, custom made, cutoff 100 kDa, membrane length 10 mm, CMA Microdialysis AB, Stockholm, Sweden) was introduced into the cubital or cephalic vein, based on visual inspection of the diameter and course, through the venous catheter. Patients were monitored for signs of complications related to the microdialysis catheters, including thrombosis of the upper extremity.

### Blood samples

Blood samples and arterial blood gases were drawn from the radial artery catheter. Additionally, blood samples from the cubital vein were collected on postoperative days 1–3 at 6 am for the analysis of inflammatory markers. Serum samples for cytokine analysis were stored at −80°C.

### Cytokine analysis

Cytokine levels were analyzed using a Bio‐Plex Magnetic Human Cytokine Panel (Bio‐Rad) on a Luminex 200 with Bio‐Plex Manager software version 6, adhering to the manufacturers' instructions. We measured the following cytokines: IL‐1 *β*, IL‐2, IL‐4, IL‐5, IL‐6, IL‐7, IL‐8, IL‐10, IL‐12 (p70), IL‐13, IL‐17, G‐CSF, GM‐CSF, IFN‐*γ*, MCP‐1 (MCAF), MIP‐1*α*, MIP‐1*β*, and TNF‐*α*. The limits of quantification are presented in Supplementary Table E1. Cytokines with concentrations below the limit of quantification in more than half of the samples were excluded from further analysis.

### Correlations

The measured cytokine concentrations were correlated with patient characteristics (age, BMI, smoke status, aortic aneurysm diameter), medication (acetylsalicylic acid, statin), comorbidities (hypertension, coronary heart disease, previous coronary intervention, heart failure, peripheral atherosclerotic disease, COPD, kidney disease, liver disease, diabetes mellitus), biochemical markers (s‐creatinine, glomerular filtration ratio), hospital admission (hospital days, intensive care unit days, anesthesia time, surgery time, aortic clamp time, intraoperative fluid volume, transfusion volume), intraoperative parameters (HR, MAP, CVP, MPAP, SVO_2_, FiO_2_/PaO_2_, temperature).

Only variables where half or more than half of the measured cytokines showed a significant correlation are presented in the Results.

### Statistics

All values are presented as the median (min–max), unless otherwise noted. To assess changes within the groups over time, we used the Friedman test. The Wilcoxon signed rank test with Bonferroni correction for multiple testing was used to compare different time points within groups. The Mann–Whitney *U* test was used to compare groups. Spearman's *ρ* was used to identify correlations. A correlation coefficient in the interval 0–0.19 was regarded as very weak, 0.2–0.39 as weak, 0.40–0.59 as moderate, 0.6–0.79 as strong, and 0.8–1 as very strong correlation. Fisher's exact test was used for binominal data. SPSS for Mac 19 (Chicago, IL, USA) was used for the statistical analyses.

## Results

There were no registered complications or adverse events due to the insertion of the microdialysis catheter or collection of bronchial ELF. The introduction of the BMD catheter into the bronchi took approximately 30 sec, and the bronchoscopy control lasted for 2 min. In four of the patients, the catheter required repositioning and one patient was excluded before the start of the experimental protocol due to a nonfunctioning microdialysis catheter.

All patients survived the 30‐day observation period after surgery. Three patients experienced severe complications that persisted >24 h after the surgical procedures requiring treatment in the intensive care unit (organ failure group). Two of these patients had unstable circulation and were fluid and norepinephrine dependent, and one patient developed postoperative renal failure. They were all three in need of ventilator support, and the length in stay in the intensive care unit was 1–11 days. Hemodynamic, respiratory, and temperature data from the perioperative period are presented in Table 3.

**Table 3:** Hemodynamic, respiratory, and temperature data; median (min–max)

	After anesthesia induction	Ischemia	Reperfusion 0–60 min	Reperfusion 60–120 min	*P*
Heart rate (beats/min)	59 (49–91)	53 (45–94)	61 (51–107)^b^	61 (51–110)^b^	0.040
Mean arterial pressure (mmHg)	82 (76–95)	72 (67–83)^a^	73 (64–91)^a^	77 (67–88)^a^	<0.001
Cardiac index (L/min/m^2^)	2.4 (1.6–3.4)	2.1 (1.5–2.4)	2.6 (1.7–3.4)^b^	2.7 (1.7–3.3)^b^	0.030
Central venous oxygen saturation (%)	71 (68–84)	67 (63–79)^a^	72 (63–81)^a^	74 (58–82)	0.001
Central venous pressure (mmHg)	10 (7–17)	9 (5–17)	11 (7–20)^b^	9 (3–17)^c^	<0.001
Mean pulmonary artery pressure (mmHg)	30 (24–36)	32 (26–35)	37 (31–56)^a^, ^b^	36 (32–43)^a^, ^b^	<0.001
PaO_2_/FiO_2_ ratio	229 (142–438)	284 (189–426)	369 (207–442)	261 (235–349)	0.237
Tidal volume (mL)/predicted weight (kg)	6.5 (4.4–8.5)	6.1 (3.4–8.2)	6.4 (4.3–8.7)	7 (5–8.6)	0.042
Lung compliance (mL/cmH_2_O)	36 (26–59)	30 (18–42)	36 (20–49)	41 (24–53)^b^	0.006
Temperature (°C)	35.9 (35.2–36.4)	35.7 (34.6–36.5)	36.1 (35.1–36.9)	36.1 (35.5–37.4)^b^	0.008

Lung volumes in the individual patient are reflected by the predicted body weight. The predicted body weights of male patients = 50 + (0.91 [height (cm) − 152.4]) and of female patients = 45.5 + (0.91 [height (cm) − 152.4]) (The Acute Respiratory Distress Syndrome Network 2000).

a *P < *0.05 versus after anesthesia induction.

b *P < *0.05 versus ischemia.

c *P < *0.05 versus reperfusion 0–60 min.

### Cytokines

#### Bronchial cytokines measured by microdialysis

Eight of 18 cytokines measured in bronchial ELF presented an overall change in relation to the surgery. IL‐5, IL‐6, IL‐13, GM‐CSF, IL‐2, IL‐4, and TNF‐*α* increased, with the highest concentrations observed during reperfusion. IL‐6 increased more than 10‐fold and IL‐13 more than 5‐fold. IL‐7 was reduced about threefold during ischemia and reperfusion (Table 4).

**Table 4:** Bronchial cytokines as measured by microdialysis (pg/ml); median (25th percentile–75th percentile)

	After anesthesia induction	Ischemia	Reperfusion 60 min	Reperfusion 120 min	*P*
IL‐1*β*	1.89 (0.77–2.55)	0.91 (0.51–6.85)	1.19 (0.48–13.83)	1.86 (0.76–14.49)	0.073
IL‐2	0.42 (0.25–0.58)	0.46 (0.25–1.06)	0.69 (0.44–1.31)	0.54 (0.35–1.28)	0.045
IL‐4	0.19 (0.11–0.35)	0.2 (0.14–0.49)	0.26 (0.2–0.69)	0.29 (0.15–0.53)	0.025
IL‐5	0.2 (0.09–0.29)	0.14 (0.09–0.23)	0.21 (0.13–0.42)	0.32 (0.12–0.67)^b^	0.012
IL‐6	18.31 (7.86–58.84)	87.77 (27.61–295.73)	229.39 (93.97–594.83)^a^	203.04 (49.08–571.83)^a^	<0.001
IL‐7	1.12 (0.78–1.73)	0.44 (0.3–0.91)^a^	0.64 (0.25–0.77)	0.47 (0.24–0.56)^a^	0.002
IL‐8	1139.39 (233.69–1512.41)	1220.95 (107.85–1740.56)	1442.42 (189.66–2051.8)	941.71 (295.58–1956.36)	0.212
IL‐10	0.35 (0.25–0.43)	0.49 (0.31–1.33)	0.56 (0.31–2.25)	0.54 (0.26–1.4)	0.115
IL‐12 (p70)	0.73 (0.47–0.97)	0.57 (0.36–1.13)	0.86 (0.47–1.36)	0.69 (0.41–1.62)	0.508
IL‐13	0.12 (0.08–0.34)	0.38 (0.11–0.63)	0.64 (0.32–2.08)^a^	0.81 (0.14–2.7)^a^	0.001
IL‐17	4.15 (1.53–7.39)	4.6 (2.76–13.55)	4.81 (3.55–17.81)	4.35 (2.25–18.2)	0.154
G‐CSF	24.35 (1.84–73.36)	62.37 (8.31–217.48)	129.59 (6.87–250.8)	70.95 (4.28–107.07)	0.145
GM‐CSF	10.11 (8.62–11.75)	11.7 (9.94–13.6)	12.7 (10.87–15.72)^a^	13.63 (10.3–15.77)^a^	0.008
IFN‐*γ*	4.55 (0.25–14.61)	9.05 (0–21.37)	11.4 (1.89–27.34)	11.01 (0–27.06)	0.337
MCP‐1	184.65 (63.98–457.85)	248.81 (78.37–613.97)	409.84 (89.86–634.08)	194.55 (68.53–577.59)	0.290
MIP‐1*α*	6.52 (3.49–19.85)	6.88 (2.22–129.98)	5.26 (2.43–147.19)	3.62 (2.1–123.23)	0.272
MIP‐1*β*	125.15 (48.7–245.54)	131.37 (48.51–445.66)	94.05 (51.36–417.3)	74.82 (28.6–418.1)	0.318
TNF‐*α*	1.39 (0.99–2.78)	1.99 (0.82–15.3)	3.71 (1.31–23.09)	2.52 (1.05–16.58)	0.038

a *P* < 0.05 versus after anesthesia induction.

b *P* < 0.05 versus ischemia.

*P* < 0.05 versus reperfusion 60 min.

More than half of the measured bronchial cytokines showed a significant correlation with the aortic aneurysm diameter, s‐creatinine, GFR, previous coronary intervention, kidney disease, statin use, duration of cross‐clamping, duration of surgery, duration of anesthesia, intensive care days, volume of erythrocyte transfusion, and MPAP. The correlation coefficients are presented in Supplementary Table E2.

The concentrations of bronchial cytokines as measured by microdialysis were significantly higher than the concentrations measured in blood by VMD.

#### Venous cytokines measured by microdialysis

Eleven cytokines measured in venous blood presented an overall change in relation to surgery. IL‐1*β*, IL‐4, IL‐6, IL‐8, IL‐10, IL‐12 (p70), IL‐17, MCP‐1, MIP‐1*α*, MIP‐1*β*, and TNF‐*α* increased, with the highest concentrations observed during reperfusion. TNF‐*α*, MIP‐1*α*, and MIP‐1*β* increased during ischemia. All cytokines increased fivefold or more except IL‐10 (Table 5). Six of the cytokines measured in venous blood (IL‐2, IL‐5, IL‐7, IL‐13, G‐CSF, and IFN‐*γ*) were below the limit of quantification in more than half of the microdialysis samples.

**Table 5:** Venous cytokines as measured by microdialysis (pg/ml); median (25th percentile–75th percentile)

	After Anesthesia Induction	Ischemia	Reperfusion 60 min	Reperfusion 120 min	*P*
IL‐1*β*	0.02 (0.01–0.04)	0.1 (0.06–0.17)	0.26 (0.16–0.48)^a^	0.7 (0.38–1.08)^a^, ^b^	<0.001
IL‐4	0 (0–0.03)	0.05 (0–0.1)	0.1 (0.05–0.16)^a^	0.12 (0.05–0.18)^a^	<0.001
IL‐6	0.27 (0.05–2.61)	17.2 (0.72–60.63)	55.1 (3.62–111.23)^a^, ^b^	42.34 (4.14–124.04)^a^, ^b^	<0.001
IL‐8	0.62 (0.15–4.95)	18.8 (1.14–47.81)	47.74 (5.92–111.43)^a^	174.84 (26.68–203.18)^a^, ^b^	<0.001
IL‐10	0.2 (0.16–0.23)	0.26 (0.2–0.33)	0.25 (0.2–0.32)	0.29 (0.23–0.37)^a^	0.013
IL‐12 (p70)	0.07 (0–0.35)	0.33 (0.2–0.73)	0.58 (0.28–1.08)^a^	0.52 (0.24–0.74)^a^	<0.001
IL‐17	0 (0–0.75)	1.13 (0–3.42)	2.75 (0.38–4.19)^a^	1.85 (0.25–3.59)^a^	<0.001
GM‐CSF	8.72 (7.5–10.03)	9.47 (7.92–11.43)	10.38 (9.26–10.63)	10.09 (8.88–11.8)	0.064
MCP‐1	8.21 (3.9–10.29)	30.01 (4.92–111.6)	78.93 (12.04–166.76)^a^, ^b^	99.5 (28.17–183.88)^a^, ^b^	<0.001
MIP‐1*α*	1.19 (0–4.61)	10.43 (1.8–16.39)^a^	7.6 (3.87–14.45)^a^	6.97 (3.86–14.28)^a^	<0.001
MIP‐1*β*	6.16 (2.24–32.63)	51.9 (13.16–150.97)^a^	61.36 (34.99–146.87)^a^	71.9 (40.37–137.24)^a^	<0.001
TNF‐*α*	0.2 (0.02–0.64)	0.89 (0.64–1.64)^a^	0.93 (0.37–1.62)^a^	0.98 (0.6–1.31)^a^	<0.001

a *P* < 0.05 versus after anesthesia induction.

b *P* < 0.05 versus ischemia.

*P* < 0.05 versus reperfusion 60 min.

More than half of the venous cytokines showed a significant correlation with age, aortic aneurysm diameter, kidney disease, acetylsalicylic acid use, smoking status, duration of cross‐clamping, duration of surgery, duration of anesthesia, volume of erythrocyte transfusion, volume of other fluids, MPAP, lung compliance, temperature, duration of stay, and number of organ failures (Supplementary Table E2).

#### Serum cytokines

Fifteen cytokines presented an overall change in relation to surgery. The highest concentration of IL‐1*β*, IL‐4, IL‐5, IL‐7, IL‐12 (p70), IL‐13, IFN‐*γ*, and TNF‐*α* was observed in the sample collected immediately after anesthesia induction. The highest concentration of IL‐8, IL‐10, G‐CSF, MCP‐1, and MIP‐1*β* was observed during reperfusion. IL‐6 peaked on the morning of the first postoperative day, and the lowest MIP‐1*α* concentration was observed on postoperative day 1. G‐CSF and MCP‐1 had a fourfold or larger increase. (Table 6). IL‐2, IL‐17, and GM‐CSF were below the limit of quantification in more than half of the samples.

**Table 6:** Serum cytokines (pg/mL); median (25th percentile–75th percentile)

	After Anesthesia Induction	Ischemia	Reperfusion 60 min	Reperfusion 120 min	Postop day 1	Postop day 2	Postop day 3	*P*
IL‐1*β*	0.97 (0.76–1.21)	0.52 (0.29–0.75)^a^	0.66 (0.46–0.78)^a^	0.83 (0.65–1.06)	0.56 (0.37–0.84)^a^	0.66 (0.5–1.04)	0.77 (0.57–1.16)	0.003
IL‐4	1.23 (0.97–1.67)	0.49 (0.28–0.94)^a^	0.83 (0.55–1.39)	1.02 (0.74–1.36)	0.64 (0.39–0.83)^a^, ^d^	0.9 (0.54–1.14)	0.88 (0.39–1.61)	<0.001
IL‐5	1.46 (1.13–1.7)	0.75 (0.26–0.93)	0.89 (0.43–1.18)	1.2 (0.79–1.62)	0.73 (0.49–1.25)^a^	0.91 (0.5–1.21)	0.96 (0.49–2)	0.011
IL‐6	2.46 (1.75–3.47)	12.14 (7.16–18.46)	26.72 (17.48–47.59)^a^	51.95 (30.92–90.75)^a^, ^b^	80.26 (49.56–137.01)^a^, ^b^	44.69 (35.92–98.8)^a^, ^b^	21.67 (11.29–53.33)^a^, ^e^	<0.001
IL‐7	7.14 (6.01–9.02)	4.03 (2.01–6.02)	4.96 (3.1–6.34)^a^	6.15 (3.81–7.03)	4.43 (3.1–5.36)^a^	4.48 (4.07–5.83)	4.7 (4.01–9.35)	0.002
IL‐8	8.11 (5.99–9.39)	7.94 (5.09–12.99)	11.55 (6.77–18.17)	19.37 (14.16–33.47)^a^, ^b^, ^c^	14.17 (11.11–18.01)^b^	13.35 (8.73–18.34)^b^	10.02 (7.56–16.89)	<0.001
IL‐10	1.02 (0.51–1.74)	0.76 (0.42–1.24)	2.6 (1.01–4.63)	2.88 (1.95–9.24)^a^, ^b^	1.71 (0.75–2.37)	1.27 (0.52–2.06)	1.24 (0.01–2.42)^d^	0.001
IL‐12 (p70)	6.68 (3.64–10.17)	1.71 (1.42–4.47)^a^	3.41 (1.65–5.34)^a^	5.04 (3.78–6.64)	3.01 (1.8–4.46)^a^	2.98 (1.97–4.22)^a^	3.38 (2.12–6.36)	<0.001
IL‐13	1.93 (1.23–2.56)	0.72 (0.01–1.22)	0.84 (0.55–1.32)^a^	1.37 (0.89–1.7)	0.81 (0.45–1.32)^a^	0.9 (0.5–1.58)^a^	1.31 (0.9–1.85)	<0.001
G‐CSF	7.34 (4.78–10.24)	6.02 (2.28–9.43)	15.77 (4.33–18.79)	30.66 (21.48–49.09)^a^, ^b^	6.84 (5.07–12.72)^d^	9.34 (3.71–17.54)^d^	8.72 (2.28–22.24)^d^	<0.001
IFN‐*γ*	59.97 (39.76–85.55)	18.63 (0–30.93)^a^	28.44 (7.9–44.45)^a^	33.77 (25.53–60.72)	24.69 (6.1–43.23)^a^	25.11 (9.13–45.39)	37.79 (7.3–73.31)	<0.001
MCP‐1	28.18 (15.06–31.89)	62.09 (38.78–126.19)^a^	154.67 (71.08–212.98)^a^	187.75 (84.88–364.05)^a^	54.08 (30.71–86.85)^a^, ^d^	45.49 (29.89–73.7)^d^	39.4 (22.36–44.39)^c^, ^d^	<0.001
MIP‐1*α*	1.54 (1.18–2.29)	0.63 (0–1.01)	1.17 (0.33–1.88)	1.74 (0.98–2.24)	0.67 (0.22–0.99)^a^, ^d^	1.09 (0.55–1.59)	1.12 (0.28–2.03)	<0.001
MIP‐1*β*	77.57 (48.99–123.09)	86.69 (54.95–123.39)	121.57 (81.08–176.86)	150.84 (115.68–199.33)^a^, ^b^	85.84 (60.85–95.77)^d^	70.37 (34.15–105.16)^c^, ^d^	84.85 (32.99–96.28)^c^, ^d^	<0.001
TNF‐*α*	13.16 (9.97–15.49)	6.18 (2.27–10.41)^a^	9.25 (4.46–11.92)^a^	11.63 (8.07–13.88)	7.25 (4.46–10.62)^a^	8.39 (5.13–13.08)	7.82 (4.7–14.22)	0.001

a *P* < 0.05 versus after anesthesia induction.

b *P <* 0.05 versus ischemia.

c *P < *0.05 versus reperfusion 60 min.

d *P *< 0.05 versus reperfusion 120 min.

e *P *< 0.05 versus 1. Postop day.

Half and more of the measured serum cytokines correlated with acetylsalicylic acid use and MAP (Supplementary Table E2).

### Perioperative organ failure; comparison of groups

Seventeen of 18 bronchial ELF cytokines (IL‐1*β*, IL‐2, IL‐4, IL‐5, IL‐6, IL‐8, IL‐10, IL‐12 (p70), IL‐13, IL‐17, G‐CSF, GM‐CSF, IFN‐*γ*, MCP‐1, MIP‐1*α*, MIP‐1*β*, and TNF‐*α*) were significantly higher in the organ failure group compared to the nonorgan failure group. For comparison, only four cytokines (IL‐4, IL‐17, MCP‐1, and MIP‐1) measured in venous blood were significantly higher in patients with organ failure comparing patients without such complications and seven cytokines measured in serum were significantly different between these two groups. The data on group comparisons are presented in Table 7.

**Table 7:** Comparison of cytokines between the organ failure group and the nonorgan failure group

	Nonorgan failure	Organ failure	*P*
(a) Bronchial cytokines (pg/ml) median (25% quartile–75% quartile)			
IL‐1*β*	1 (0.68–3.61)	9.67 (1.78–30.28)	0.003
IL‐2	0.44 (0.26–0.71)	1.07 (0.81–1.93)	0.002
IL‐4	0.2 (0.14–0.31)	0.52 (0.34–0.99)	<0.001
IL‐5	0.16 (0.1–0.31)	0.41 (0.27–0.62)	0.021
IL‐6	82.96 (20.42–223.08)	528.62 (112.3–859.25)	0.003
IL‐7	0.51 (0.26–0.77)	0.91 (0.6–1.2)	0.074
IL‐8	967.36 (273–1511.77)	1904.31 (1386.32–2657.53)	0.001
IL‐10	0.35 (0.27–0.66)	1.63 (0.47–3.71)	0.001
IL‐12	0.59 (0.41–0.93)	1.38 (0.79–2)	0.001
IL‐13	0.36 (0.08–0.66)	0.85 (0.37–3.62)	0.034
IL‐17	4.28 (2.55–7.72)	13.29 (5.91–24.61)	0.001
G‐CSF	26.79 (6.87–103.55)	220.65 (73.76–279.32)	0.001
GM‐CSF	11.33 (9.9–13.39)	16.19 (12.01–21.56)	0.017
IFN‐*γ*	5.64 (0–12.27)	36.89 (15.73–58.31)	<0.001
MCP‐1	184.65 (79.18–518.36)	632.01 (229.14–748.15)	0.014
MIP‐1*α*	5.96 (2.77–14.02)	28.59 (8.95–137.17)	0.001
MIP‐1*β*	74.84 (49.93–225.75)	347.9 (210.56–484.98)	0.037
TNF‐*α*	1.71 (0.93–3.94)	11.96 (3.08–35.1)	0.016
(b) Venous cytokines (pg/ml) median (25% quartile–75% quartile)			
IL‐1*β*	0.11 (0.04–0.39)	0.38 (0.07–0.9)	0.253
IL‐4	0.04 (0–0.11)	0.14 (0.05–0.22)	0.019
IL‐6	6.21 (0.49–66.16)	31.27 (4.5–121.44)	0.242
IL‐8	15.73 (1.25–73.16)	57.21 (19.56–163.37)	0.126
IL‐10	0.23 (0.17–0.3)	0.29 (0.23–0.46)	0.278
IL‐12	0.28 (0.08–0.6)	0.62 (0.43–0.82)	0.074
IL‐17	0.58 (0–2.47)	3.56 (2.17–6.56)	0.037
GM‐CSF	9.66 (8.19–10.88)	10.56 (9.6–12.65)	0.904
MCP‐1	25.92 (7.67–97.61)	103.54 (24.49–219.58)	0.046
MIP‐1*α*	5.09 (1.8–10.92)	8.58 (5.5–15.69)	0.203
MIP‐1*β*	40.68 (13.16–76.72)	145.45 (66.88–155.4)	0.135
TNF‐*α*	0.67 (0.2–1.31)	0.98 (0.73–1.28)	0.003
(c) Serum cytokines (pg/ml) median (25% quartile–75% quartile)			
IL‐1*β*	0.67 (0.46–0.96)	1.05 (0.7–1.22)	0.006
IL‐4	0.87 (0.52–1.26)	1.27 (0.88–1.77)	0.042
IL‐5	0.97 (0.62–1.41)	1.46 (0.91–1.65)	0.072
IL‐6	17.55 (3.81–42.23)	38.6 (7.37–52.43)	0.174
IL‐7	5.38 (3.65–6.83)	6.7 (5.63–7.36)	0.108
IL‐8	9.34 (6.16–16.54)	13.95 (10.64–16.18)	0.076
IL‐10	1.4 (0.8–2.65)	2.11 (0.39–9.84)	0.470
IL‐12	3.91 (1.67–6.58)	5.81 (4.19–9.29)	0.102
IL‐13	1.15 (0.62–1.66)	1.37 (1.12–1.86)	0.414
G‐CSF	9.71 (5.36–20.76)	12.97 (8.44–22.91)	0.409
IFN‐*γ*	31.34 (17.74–56.81)	54.36 (27.41–74.55)	0.153
MCP‐1	74.89 (31.84–173.15)	109.64 (38.38–282.44)	0.449
MIP‐1*α*	1.12 (0.56–1.73)	2.33 (1.25–2.92)	0.051
MIP‐1*β*	95.22 (66.52–135.11)	171.58 (107.67–274.85)	0.005
TNF‐*α*	9.61 (6.15–13.12)	12 (11.13–14.8)	0.001

All measured cytokine concentrations at after anesthesia induction, ischemia, reperfusion 60 min and reperfusion 120 min are included.

## Discussion

In this study, we show that BMD is a safe procedure in mechanically ventilated patients. We found higher cytokine levels in ELF obtained by BMD than in venous blood and serum, and we demonstrated significantly changes in several of these cytokines both peri‐ and postoperatively in patients undergoing elective open AAA repair. In particular, we show that the IL‐6 levels were increased more than 10‐fold and IL‐13 levels more than fivefold in ELF during ischemia and reperfusion. Also, the venous levels of several inflammatory and anti‐inflammatory cytokines increased and exhibited their highest concentration during reperfusion. Both bronchial and venous cytokine levels correlated with duration of the procedure, intensive care days, and preoperative kidney disease. Three patients suffered organ failure as a direct consequence of the procedure, and in these patients the bronchial ELF concentrations of seventeen of 18 cytokines differed significantly from patients without such complications. Although this is a small study, we show significantly higher cytokine levels in ELF from patients developing perioperative organ failures comparing those without such complications.

This pilot study support a role for BMD in monitoring patients during vascular surgery, but clearly, larger studies are needed to evaluate its power as a predictive tool for complications in these patients. The gold standard for monitoring of the ELF is bronchoalveolar lavage (BAL). However, measurements in ELF from BAL only represent a snapshot. Continuous monitoring or several repeated measurements is needed to better describe inflammatory responses in the lungs. BAL is relatively traumatic, and is often accompanied by adverse events (De Pascale et al. 2015). BMD is safe with no adverse events, provides possibilities for repeated measurements/continuous monitoring, and can most probably be used in intubated critically ill patients intubated over time.

Using BMD we detected 18 different cytokines in the ELF, and several of these cytokines changed significantly during reperfusion. In particular, IL‐6 increased more than 10‐fold and IL‐13 more than 5‐fold. These two cytokines could be particularly suitable as biomarkers for pulmonary inflammation as they also reflect the pathogenesis of acute lung injury and repair. First, elevated levels of IL‐6 in plasma and BAL have been shown in several studies to predict mortality in acute lung injury (Meduri et al. 1995; Parsons et al. 2005). It has also been shown that IL‐6 may be a useful biomarker for stratification of asthma patients by reflecting poor lung function and increased risk for developing exacerbations (Peters et al., 2016). IL‐13 could also potentially reflect an interesting biomarker for pulmonary inflammation and injury. Thus, IL‐13 may induce many features of allergic lung disease, and has also been implicated in the pathogenesis of acute lung inflammatory injury (Baurakiades et al. 2014). Recently, Chung et al. (2016) showed that IL‐13 is a major regulator of radiation‐induced lung injury and that therapeutic neutralization of IL‐13 was protective against lung fibrosis. Accordingly, our findings of a marked increase of IL‐6 and IL‐13 in ELF could potentially be novel biomarkers for lung complications during vascular surgery. However, before ELF levels of IL‐6 and IL‐13 may be used as biomarkers, future studies will have to demonstrate that the ELF levels of these cytokines has high sensitivity and specificity in predicting clinical outcome, and can be measured with reproducible results across multiple clinical settings and sites.

The concentrations of bronchial cytokines were higher than the concentrations measured by VMD, supporting the hypothesis that the cytokines measured in bronchi are produced within the lung and bronchi and do not only reflect a spillover from a systemic response. One of the challenges with cytokine sampling by microdialysis is the low relative recovery for cytokine sampling by microdialysis. Some important factors as molecule size, diffusion coefficient (physical and chemical properties of the cytokine), and perfusion fluid velocity of the microdialysis pump affects recovery (Waelgaard et al. 2006; Helmy et al. 2009). This study was not designed to compare concentrations of cytokines in arterial serum samples and in microdialysate. But in view of our data with substantial higher concentrations measured in the bronchi compared to the venous circulation, we suggest that the production of cytokines is compartmentalized, and that the bronchial cytokines are produced in the lung in response to the surgical trauma from open AAA repair.

Our findings in intermittent serum samples were unlike previously reported results and unlike our findings with continuous venous blood sampling using VMD. In serum samples in the first time periods (after anesthesia induction, ischemia, reperfusion 60 min, reperfusion 120 min), we saw both proinflammatory increase (IL‐6) and decrease (IL‐1*β*, IL‐7, IL‐12(p70), IL‐13, and TNF‐*α*). Chemokines increased (IL‐8, MCP‐1, and MIP‐1*β*). Inhibitory cytokines both decreased (IL‐4 and IFN‐*γ*) and increased (IL‐10). This decrease in several serum cytokines during the ischemia and reperfusion periods may reflect a hyperacute release of cytokines at the time of the “after anesthesia induction” serum sample. The optimal time point for baseline cytokine samples in open AAA repair is unclear, and the results from different studies cannot be compared directly (Roumen et al. 1993; Barry et al. 1997; Groeneveld et al. 1997; Lammers et al. 2003). Studies on the timing of the baseline blood sample are required to clarify the possible effects of presurgical procedures on the day of surgery.

Both BMD and VMD correlated with premorbid status, procedural factors, and parameters known to impact outcome (Supplementary Tables E2a and E2b) (Brady et al. 2000). The correlations were mostly weak to moderate with only a few strong correlations. With the high number of correlations there is a risk that there are false positive and false negative in our material. The presentation of the correlations bear this in mind and only present factors where more than half of the cytokines showed a significant correlation, and consider only these factors to be significant. The overall cytokine concentrations in the bronchial and venous microdialysate correlated well with historically useful parameters for predicting outcome in patients undergoing elective open AAA repair. This indicate that bronchial and venous cytokines are biochemical markers correlating well with the trauma of open AAA repair, and that microdialysis is a reliable method for monitoring the inflammatory response.

The serum samples only correlated with acetylic acid use and MAP (Supplementary Table E2c). It is necessary to evaluate the timing of intermittent samples. Sampling more frequently may provide more reliable data.

The study was not powered to compare groups of patients based on outcome variables. However, the bronchial cytokine response was different in patients suffering from severe early complications, with duration of organ failure more than 24 h that were only caused by the trauma of open AAA repair. The organ failure group demonstrated a significant change at the concentrations of 17 of 18 bronchial cytokines within 120 min of reperfusion after open AAA repair when compared to the nonorgan failure group. The bronchial cytokines measured during and 2 h after open AAA repair distinguished patients with a postoperative course including organ failure caused only by the trauma from the procedure of open AAA repair and patients without organ failure according to the criteria. The differences between the groups included a generally higher bronchial cytokine concentration after anesthesia induction and an increase in the bronchial cytokine concentration during I/R. We believe that cytokine production is compartmentalized, and the concentrations measured in the blood and bronchi have different patterns (Tyvold et al. 2010). In serum samples and VMD, some cytokines show the same trend, but the response was not as consistent as in the bronchial ELF. We found that the cytokine concentrations in the bronchial ELF best identified patients who developed organ failure according to the criteria in a direct time relation to open AAA repair.

## Conclusions

We present a pilot study showing that BMD is a safe method for continuous monitoring of inflammatory responses during open AAA repair. In our study, bronchial IL‐6, IL‐13, and TNF‐*α* and venous IL‐1*β*, IL‐6, TNF‐*α*, IL12 (p70), IL‐8, MIP‐1*α*, MIP‐1*β*, and MCP‐1 inhibitory cytokines IL‐4, IL‐17 are candidates as single markers for the trauma of open AAA repair. But our data show that measuring many cytokines at the same time by microdialysis in the distal bronchi or the cubital vein better depicts the cytokine response by the trauma of open AAA repair, and that the measurement of several inflammatory cytokines in ELF may better predict the risk for immediate complications leading to organ failure in patients undergoing open AAA repair.

We need new analyzing methods that can give expedite results at the bedside to make these results relevant to the single patient undergoing open AAA repair.

Further studies are needed exploring the role for BMD and measurements of ELF cytokine levels in predicting complications and outcome for patients undergoing vascular surgery.

## Declarations

### Ethics approval and consent to participate

The manuscript was approved by the Regional Committee for Health and Medical Research (2010/1649), and written informed consent was obtained from all patients.

### Trial registration

NCT01322295. ClinicalTrials.gov 23 March 2011.

### Availability of data and material

The datasets analyzed during the current study are available from the corresponding author on reasonable request.

## Conflict of Interests

None of the authors have any conflict of interest.

## Data Accessibility
